# Blood immune profiles in chronic low back pain patients with type 1 Modic changes are associated with intradiscal propionic acid levels

**DOI:** 10.21203/rs.3.rs-10508730/v1

**Published:** 2026-08-03

**Authors:** Jan Devan, Jiamin Zhou, Po-Hung Wu, Noah Bonnheim, Conor O’Neill, Jeffrey C. Lotz, Thomas M. Link, Abel Torres-Espin, Oliver Distler, Stefan Dudli, Aaron J. Fields

**Affiliations:** University of Zurich; University of California, San Francisco; Atrium Health Musculoskeletal Institute Wake Forest University School of Medicine; University of California, San Francisco; University of California, San Francisco; University of California, San Francisco; University of California, San Francisco; University of Waterloo; University of Zurich; University of Zurich; University of California, San Francisco

**Keywords:** low back pain, B cells, Modic changes, magnetic resonance spectroscopy

## Abstract

**Background:**

Modic type 1 changes (MC1) are vertebral endplate bone marrow lesions associated with chronic low back pain (CLBP), but their underlying pathobiology remains unclear. Disc–bone marrow crosstalk that includes *Cutibacterium acnes (C. acnes)* infection of the disc and immune system activation in the adjacent vertebrae may be a defining feature of some MC1.

**Methods:**

In a prospective analysis of patients from the UCSF comeBACK cohort, we quantified intradiscal propionic acid (PA) – a metabolic product of *C. acnes* – using magnetic resonance spectroscopy (MRS). Patients were stratified into tertiles based on intradiscal PA content, and the uppermost (PA-high) and lowermost (PA-low) tertiles were compared for systemic immune signatures, including whole-blood transcriptomics (n = 196), flow cytometry immunophenotyping (n = 224), and serum cytokine profiling (n = 398).

**Results:**

High intradiscal PA was associated with distinct systemic immune responses in patients with MC1 but not in patients with Modic type 2 changes or without Modic changes. Transcriptomic analysis revealed enrichment of adaptive immune pathways and B cell activation signatures in PA-high MC1. Flow cytometry identified the expansion of immunosuppressive ectonucleotidases CD39 and CD73-expressing B cells in PA-high MC1 patients. Finally, intradiscal PA levels were correlated with circulating B cells and serum cytokine concentrations.

**Conclusions:**

Intradiscal PA levels measured by MRS associate with biologically distinct blood profiles characterised by systemic B cell activation in patients with MC1. These findings suggest a systemic immune component in the pathobiology of MC1 and highlight the potential of MRS-derived intradiscal PA levels to stratify patients with MC1-related chronic low back pain into biologically distinct subgroups.

## Background

Low back pain (LBP) is the most common musculoskeletal condition and is the leading cause of disability worldwide. The aetiology of LBP is multifactorial^1^. In some patients, LBP may be caused by spinal pathologies that appear on magnetic resonance imaging (MRI). In particular, vertebral endplate bone marrow lesions, which appear as signal intensity changes on routine clinical MRI (“Modic changes”, or MC), are significantly associated with chronic LBP (CLBP)^2,3^ and are more prevalent in CLBP patients than in asymptomatic controls^4^. Despite advances in MC-specific treatments, clinical management of CLBP in patients with MC remains challenging, as treatment outcomes are highly heterogeneous^5,6^. This heterogeneity may reflect differences in MC pathophysiology not captured by conventional MRI. Such differences in MC pathophysiology could involve differences in bone marrow activation, differences in blood immune cell composition, and differences in cytokine profile^7–10^. Identifying differences in the blood immune profile of CLBP patients with MC could, therefore, clarify MC pathophysiology, motivate diagnostic approaches that allow for better patient stratification, and inform therapeutic strategies that more precisely target the underlying pathophysiology.

**Figure 1:**
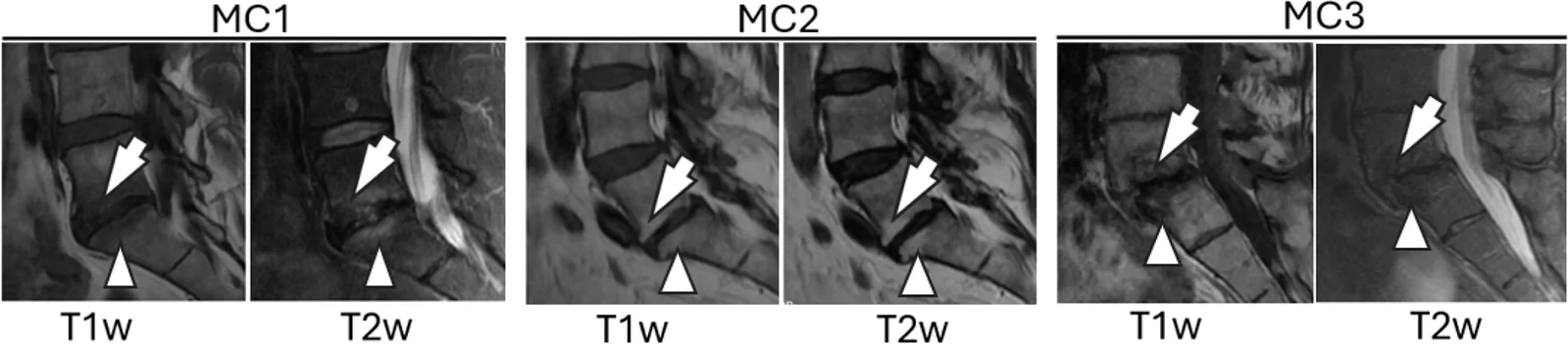
Representative T1- and T2-weighted MRI images of Modic type 1, 2 and 3 changes. MC1, Modic type 1 changes; MC2, Modic type 2 changes; MC3, Modic type 3 changes.

Current MRI assessment of MC provides little insight into pathophysiology. MC are categorized into three types based on MRI signal characteristics: type 1 changes (MC1), which are hypointense on T1-weighted (T1w) and hyperintense on T2-weigthed (T2w) sequences and reflect fibrovascular marrow replacement; type 2 changes (MC2), which are hyperintense on T1w and T2w sequences and reflect fatty marrow replacement; and type 3 changes (MC3), which are hypointense on T1w and T2w sequences and reflect endplate sclerosis (Fig. 1)^11^. However, within a single MC type, there is strong evidence of divergent pathobiology, including variation in the microbiome of MC-adjacent discs^12,13^. The most commonly implicated microbe in MC-adjacent discs is *Cutibacterium acnes (C. acnes)*^14^. For example, stratifying individuals with MC1 by the *C. acnes* burden in the adjacent disc reveals significant differences in both local and systemic immune profiles^12^. In animal models, *C. acnes* induced MC1-like marrow changes, which supports a causal role in disease pathogenesis^12,15^.

To understand the pathobiology of MC, prior studies analyzed disc biopsies collected intraoperatively during spinal fusion surgery^8,12,16^, which is the gold standard for profiling the local cell and molecular milieu. However, because this sampling procedure is limited to patients undergoing surgery for CLBP, the findings have unclear applicability to CLBP patients who are managed non-surgically and who may benefit most from pathophysiology-guided care. Also, biopsy-based profiling of the tissue carries a potential risk of contamination, raising concerns about the clinical validity of these findings. To address these challenges and allow for assessment of disc microbial and immune activity across the broader CLBP population, we aimed to discover MC pathobiology using non-invasive magnetic resonance spectroscopy (MRS) of intervertebral disc metabolites and blood immune profiling. Both methodologies are independently associated with intradiscal *C. acnes*. Specifically, MRS can quantify intradiscal levels of propionic acid (PA), a metabolite produced by *C. acnes*, and MRS-measured intradiscal PA levels correlate with disc *C. acnes* concentrations^17,18^. Separately, blood cytokine profiles differ between MC1 patients with high vs low intradiscal biopsy *C. acnes* loads^12^. We applied these non-invasive approaches to a large non-surgical cohort of CLBP patients with variable levels of intradiscal PA levels. Based on results from prior surgical cohorts, we hypothesized that CLBP patients with MC1 and high MRS-measured intradiscal PA levels have distinct blood immune profiles compared with MC1 patients with low MRS-measured intradiscal PA levels. If our hypothesis is correct and MC1 patients exhibit biologically distinct immune signatures depending on their intradiscal PA levels, this finding would have clinical implications for future pathophysiology-based patient stratification.

## Methods

This prospective study was conducted with institutional review board approval (WIRB #20-30368) and consent of the cantonal ethics commission (BASEC #2019 − 00845). Written informed consent was taken from all individual participants.

### Subjects

A subset of participants in The Longitudinal Clinical Cohort for Comprehensive Deep Phenotyping of Chronic Low-Back Pain Adults Study (comeBACK)^19^, a part of the NIH Back Pain Consortium (BACPAC)^20^, were included in this analysis. To be eligible for inclusion in the present study, comeBACK participants must have had (a) an available clinical MRI and single-voxel MRS acquisitions and (b) blood serum and blood RNA samples collected at the time of MRI and MRS. Of the 463 total participants enrolled in comeBACK, 398 patients had blood sampled at their baseline visit available for cytokine quantification and 389 for total blood transcriptomic analysis. Of those 398 patients, 200 patients underwent baseline MRI and MRS. All participants had LBP for at least three months and on at least half of the days in the past 6 months (**Fig. S1**). Patient-reported outcome measures for assessing LBP severity included the Patient-Reported Outcomes Measurement Information System (PROMIS) Physical Function Short Form 6b^21^; and PROMIS Pain Interference Short Form 4a^22^

### Imaging

Lumbar spine MRI was performed on a Discovery MR 750 3-T scanner using an 8-channel phased-array spine coil (GE Healthcare). Sagittal T_1_-weighted spin echo and fat-saturated T_2_-weighted sequences were acquired using the following parameters for T_1_-, T_2_-weighting, respectively: TE = 15, 60 ms; TR = 511, 4788 ms; FOV = 26.0 cm; slice thickness = 3.0 mm; and in-plane resolution = 0.5 mm. A board-certified musculoskeletal radiologist (> 30 years of experience) evaluated the T_1_- and T_2_-weighted images according to a comprehensive MRI grading system^23^. In particular, MC type was scored for each endplate in the lumbar spine (L1 through S1) based on the dominant type according to standard MC definitions: type 1 MC (MC1) were hypointense on T_1_- and hyperintense on fat-sat T_2_ images; type 2 MC (MC2) were hyperintense on T_1_- and hypointense on fat-sat T_2_-weighted images.

Patients presenting with MC1 and MC2 at different lumbar endplates or within the same endplate (so-called “mixed” MC) were categorised as MC1, reflecting the more clinically significant MC type with increased inflammation and association with pain^24,25^. Since MC height and MC area have inconsistent effects on LBP outcomes, we characterized each patient according to MC presence/absence rather than MC size^26,27^.

### Magnetic resonance spectroscopy

Single-voxel MRS spectra were acquired on a Siemens Skyra 3-T scanner using a Point-RESolved Spectroscopy (PRESS) sequence and Chemical Selective Suppression (CHESS) for water suppression^17^. Briefly, shortened T_1_-weighted and T_2_-weighted acquisitions in the sagittal, coronal, and axial planes enabled voxel prescription to encompass the disc nucleus pulposus while excluding the vertebral body. Shimming was performed to optimize the water signal before initiating the MRS acquisition series of 160–192 frames using a 16-step phase cycle (1500 ms TR, 32 ms TE). Disc levels for MRS were selected by the scanner operator, who chose up to four lumbar levels per patient and acquired the spectra in a caudal-to-cranial order, blinded to the radiologist’s MC diagnoses. Acquired spectra were post-processed using the NOCISCAN-LS^™^ software (Aclarion, Inc.; Broomfield, CO), which optimized spectral quality by correcting for insufficient signal-to-noise ratio, frame editing, phase and frequency shift errors, baseline, and artefacts.

To estimate the relative amount of intradiscal *C. acnes*, the area under the spectral curve (AUC) of the propionic acid (PA) peak (chemical shift ~ 1.05 ppm) was normalized to the MRS voxel size (**Fig. S2**). In a study of bovine coccygeal discs, *C. acnes-* and PA-injected discs had a clear spectral peak corresponding to the chemical shift of PA, and the signal intensity of that PA peak depended on the amount of *C. acnes* in the inoculant^18^. Separately, we confirmed the sensitivity of MRS-derived PA measures to different PA concentrations in phantoms containing de-ionized water using the same MRS sequence parameters and MRI scanner as in the present study (**Fig. S3**).

For each patient, the maximum PA level across all sampled discs was used as a pragmatic patient-level proxy of the highest local bacterial burden, and tested for association with circulating biomarkers. Patients were then stratified by their maximum intradiscal PA values into PA tertiles: “PA-low” (little or no intradiscal bacteria likely present); “intermediate PA” (uncertain bacterial presence); and “PA-high” (higher amount of intradiscal bacteria likely present). Sensitivity analyses using the mean PA value, rather than the maximum, yielded similar patient stratification, suggesting our results are unaffected by the choice of this summary metric.

### Blood sample collection

Whole blood samples from patients were collected in the morning after imaging acquisition in one 10 mL serum separating tube for serum analysis and one 2.5 mL PAXgene blood RNA tube for RNA analysis^28^. Serum samples were allowed to clot at room temperature for 30 minutes and then centrifuged for 15 minutes at 1300 rcf at 4°C. The resulting serum supernatant was aliquoted into labelled cryovials and frozen at −80°C for storage. For RNA PAXgene analysis, the PAXgene blood RNA tubes were stored upright at room temperature for 2 hours, frozen at −20°C for 24 hours, and then transferred and stored at −80°C.

### Analysis of blood samples to identify biological differences between PA-high and PA-low subjects

To characterize systemic immunobiological features associated with intradiscal PA, an integrated multi-omics analysis of blood samples was performed, combining transcriptomics, flow cytometry-based immunophenotyping, and serum cytokine profiling. A common analytical framework was applied across all three omics platforms primarily focusing on identifying biological differences between PA-high and PA-low subjects.

### Transcriptomics

RNA was isolated from PAXgene Blood RNA tubes using RNeasy Mini Kit (Qiagen) and QiaCube Connect (Qiagen). RNA libraries were prepared for each sample with RNA Integrity Number ≥ 6 and RNA concentration > 5 ng/μL using TruSeq Stranded mRNA (Illumina). The cDNA libraries were sequenced using 100 cycles on a NovaSeq X platform (Illumina) with 100 bp single-end reads to an average of 26M reads. Data were processed using SUSHI, a fully documented and reproducible pipeline for RNA sequencing data from raw data to differential expression analysis^29,30^. This includes *FastQC* to control the quality of reads, Kallisto for genome alignment and quantification of transcript abundance.

### Flow cytometry immunophenotyping of blood cells

Cells isolated by erythrocyte lysis and centrifugation of peripheral blood were cryopreserved. After thawing, they were incubated for 1 day in Roswell Park Memorial Institute (RPMI) medium supplemented with 10% heat-inactivated fetal calf serum, penicillin/streptomycin, and stable glutamine. Afterwards, cells were stained with an immunophenotyping antibody panel (antibodies, clones, fluorochromes, vendors and catalogue numbers are listed in **Tab. S1**), fixed with fixation buffer (Biolegend) and acquired on a cytometer (Cytek Aurora, five lasers). All samples were stained and analysed in one experiment. Unmixing of spectral flow cytometry data was done using SpectroFlo (v2.3; Cytek Biosciences), compensations and gating were performed in FlowJo (v10.10; BD Biosciences).

### Serum cytokine measurement

Serum samples were assayed for simultaneous quantitative measurement of 40 inflammation-associated cytokines using Quantibody^®^ Human Inflammation Array 3 (QAH-INF-3; Raybiotech, Inc.; Norcross, GA), an array-based multiplex sandwich enzyme-linked immunosorbent assay (ELISA). Missing cytokine concentrations were imputed using principal component analysis (PCA), which estimates missing values based on the PCA while minimising the influence of outliers. Missing values were assumed to be missing at random. Results were filtered to exclude values below the limit of detection (LOD) and above the maximum for each cytokine (**Tab. S2**). Afterwards, serum cytokine concentrations were log-transformed to reduce the effects of skewness in the distributions.

### Statistical analysis

Statistical analysis of patient characteristics data was performed using JMP Pro 17.0.0 (SAS Institute; Cary, NC). A two-sided *p*-value < 0.05 was considered significant, and data are reported as mean ± SD unless noted otherwise. One-way ANOVA was used to assess differences in demographics and clinical characteristics between MC type classifications. Pairwise comparisons were performed using Tukey-Kramer HSD. One-way ANOVA was also used to assess differences in maximum measured intradiscal PA values between different MC type classifications.

For blood transcriptomics, PCA) of the top 2000 features was performed to derive principal components (PCs) and to show unbiased population-specific clustering and provide a systematic view of gene differential expression. Significantly differentially expressed genes (DEG) were determined using the DESeq2 Bioconductor R package, which implements a generalised linear model with a negative binomial distribution to account for technical and biological variability. Dispersion and log2 fold change estimates were moderated using shrinkage methods to improve stability. Statistical significance was assessed using the Wald test, with p-values adjusted for multiple testing using the Benjamini-Hochberg method to control the false discovery rate (FDR). Genes were considered differentially expressed if p < 0.01 and log2 fold-change > 0.5. Gene Ontology (GO) term pathway analysis was performed using gene-set enrichment analysis via the ‘GSEA’ function, of the clusterProfiler Bioconductor R package (version 4.18.0) with FDR thresholds of 0.05^31,32^. All R functions were executed on R version 4.6.0 and Bioconductor version 3.23.

Flow cytometry data were analysed in FlowJo (Waters Corporation) and subsequently with GraphPad Prism (Siemens) using PCA, 2-way ANOVA and correlation analysis. FlowSOM clustering was performed in R (v4.3.2)^33^. Comparison of PC scores or proportions of specific immune cells between patient groups defined by PA levels was done using ordinary one-way ANOVA or Brown-Forsythe and Welch ANOVA test based on the results of Bartlett’s test for differences in standard deviations.

Statistical analysis of the serum cytokines was performed using GraphPad Prism. Spearman correlation coefficients between cytokine concentrations and PA, and between cytokine concentrations and proportions of immune cell subsets, were calculated, and hierarchical agglomerative clustering using Euclidean distance and complete linkage was used to investigate similarities in the correlation coefficients between immune cell subset proportions and intradiscal PA levels and blood plasma cytokine concentrations.

## Results

### Patient characteristics

The analysis included 398 CLBP patients from the comeBACK study with available MRS. For 200 of these patients, lumbar spine MRI data were available, allowing diagnosis of MC. The average age of these 200 patients was 51.3 ± 15.6 years, and 42.5% were male (Table 1). Patients reported an average pain rating in the week before study participation of 4.5 ± 1.9 on the Pain, Enjoyment of Life and General Activity (PEG) scale, which ranges from no pain (0) to pain as bad as you can imagine (10). On the PROMIS assessment of pain interference (PI) and physical function (PF), patients reported an average T-score of 58.5 ± 7.7 in PI and 41.8 ± 6.4 in PF, corresponding to higher average pain interference and lower average physical function than the reference general population from the United States from PROMIS.

Eighty-five of the 200 patients (42.5%) had no MC (MC0) present at any lumbar level, 80 (40%) had at least one lumbar level graded as MC1, and 35 (17.5%) had only MC2 graded along their lumbar spine. There were no significant differences in sex, PEG scores, or PROMIS pain interference and physical function scores between patients in each MC classification. Age was significantly lower in MC0 patients compared to either MC1 or MC2 patients (45.8 years *vs*. 54.5 and 57.2 years, respectively; *p* < 0.0001), and BMI was significantly higher in patients with only MC2 compared to MC0 or MC1 (28.5 kg/m^2^ *vs*. 25.4 and 26.0 kg/m^2^, respectively; *p* = 0.003).

### Measurement of intradiscal propionic acid by MRS

Intradiscal PA levels were measured with MRS in 530 total lumbar discs from 200 patients (1–4 discs/patient). The distribution of patients’ maximum PA levels ranged from 0.00 to 18.61. The maximum PA value for all patients was 3.2 ± 3.2, with a positive skewness of 2.0 and a positive kurtosis of 5.3. There was no significant difference in intradiscal PA levels between MC1, MC2 and MC0 patients (**Fig. S4**). The maximum PA level was used to stratify patients into tertiles. Patients with “PA-low” (*n* = 69) had PA levels below 1.57, and patients with “PA-high” (*n* = 63) had PA levels above 3.65. All PA groups had similar age, sex, BMI, as well as similar PEG and PROMIS scores (**Tab. S3**).

### Transcriptional signature of adaptive immune response in “PA-high” of MC1

**Figure 2:**
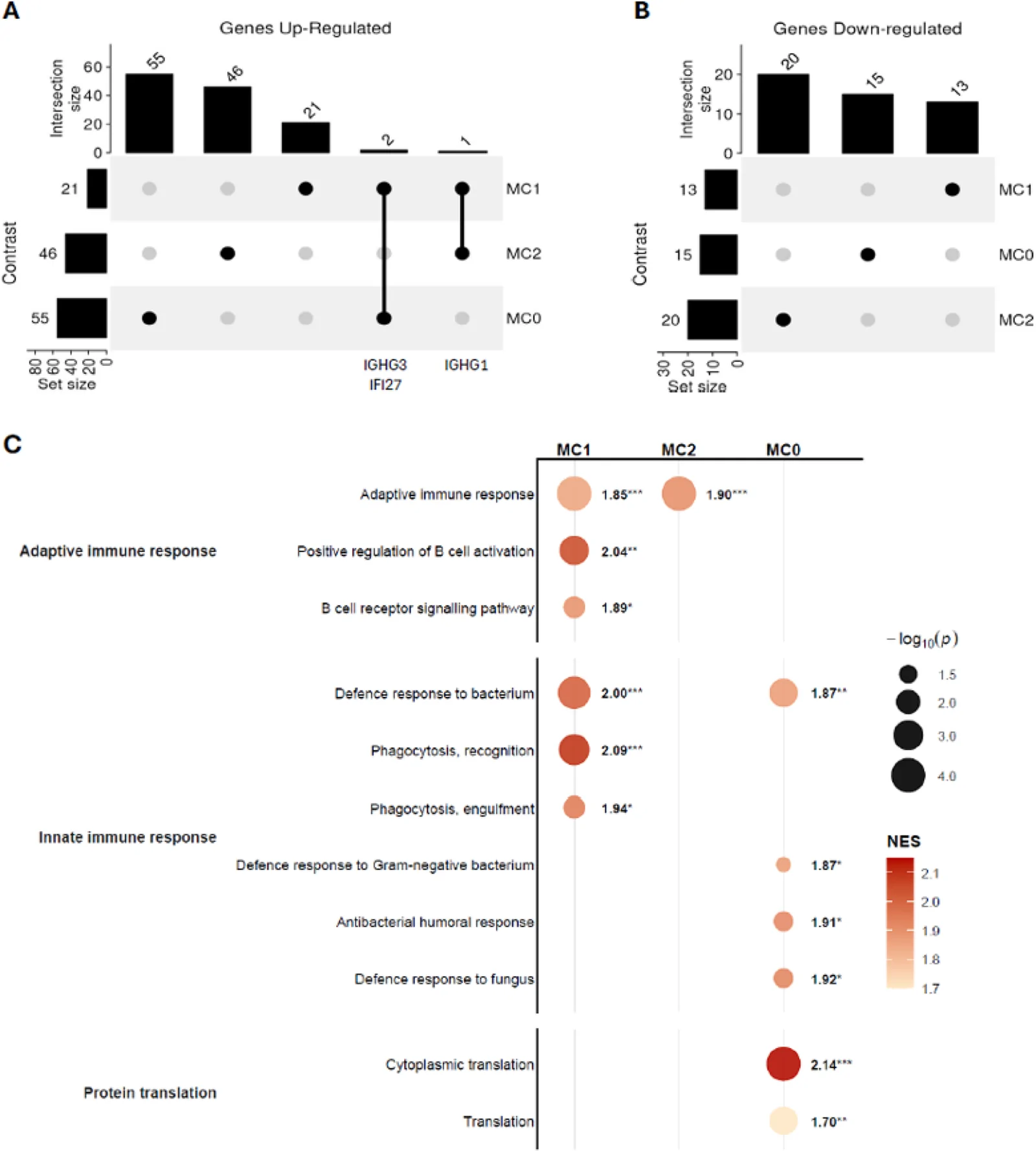
Whole blood bulk-RNA sequencing analysis from CLBP patients. (**A**) Upset plot intersecting upregulated genes in patients with “PA-high” vs. “PA-low” between MC types. Gene names are given for intersections below the plot. (**B**) Upset plot intersecting down-regulated genes in patients with “PA-high” vs. “PA-low” between MC types. (**C**) GSEA of whole-blood bulk RNA sequencing comparing patients with high pain (PA-high) and low pain (PA-low) within each Modic change subtype (MC1, MC2, and MC0). Only significantly enriched Gene Ontology (GO) biological processes are shown. Bubble colour indicates the normalized enrichment score (NES). Bubble size corresponds to statistical significance (−log10 *p* value). Numbers adjacent to bubbles indicate the NES, while asterisks denote significance (* = *P*< 0.05, ** = P < 0.01, *** = *P* < 0.001). Empty cells indicate gene sets that were not significantly enriched in the corresponding comparison.

Total blood RNA was sequenced from 196 patients; 4 did not pass the quality thresholds (RNA integrity number ≥ 6 and RNA concentration > 5 ng/μL). Eighty-four had MC1 (PA-low: 28, PA-high:27), 34 had MC2 (PA-low: 12, PA-high: 11), and 78 had MC0 (PA-low: 25, PA-high: 27). The first 10 PCs of the 2000 most variable genes explained 44.6% of total data variance (**Fig. S5A**). Samples did not cluster according to MC type (**Fig. S5B**) or PA level (**Fig. S5C**). Comparing PA-high vs PA-low in MC1, 21 genes were up- and 13 downregulated, in MC2, 46 were up- and 15 downregulated, and in MC0, 55 were up- and 20 downregulated (Fig. 2A, B). No gene was consistently up- or down-regulated in PA-high vs PA-low in all MC types. Immunoglobulin Heavy Constant Gamma (IGHG) 3 and Interferon Alpha Inducible Protein 27 (IFI27) were both upregulated in the PA-high group of MC1 and MC0. IGHG1 was upregulated in the PA-high group of MC1 and MC2. GSEA revealed enrichment of biological processes related to adaptive immune response in PA-high vs PA-low in MC1 and MC2, but not in MC0 (Fig. 2C). Activation of B cells, indicated by the GO terms “Positive regulation of B cell activation” and “B cell receptor signaling pathway” were only enriched in MC1, but not in “PA-high” of MC0 and MC2. Furthermore, innate immune processes linked to antibacterial defense mechanisms were enriched in “PA-high” vs “PA-low” in MC1 and MC0, but not in MC2 (Fig. 2C).

### Immunophenotyping by flow cytometry

Peripheral blood cells from 224 patients with CLBP were immunophenotyped using multicolour flow cytometry. Both MRS and MRI were accessible for 170 of them. The marker panel distinguished 45 main immune cell lineages and 1021 smaller cell populations corresponding to different activation or functional maturation stages of the main lineages. Samples from patients with available MC classification and intradiscal PA levels were further analysed: 73 MC1 (PA-low: 26; intermediate PA: 26; PA-high: 21), 29 MC2 (PA-low: 11; intermediate PA: 11; PA-high: 9), and 68 MC0 (PA-low: 23; intermediate PA: 22; PA-high: 23).

**Figure 3:**
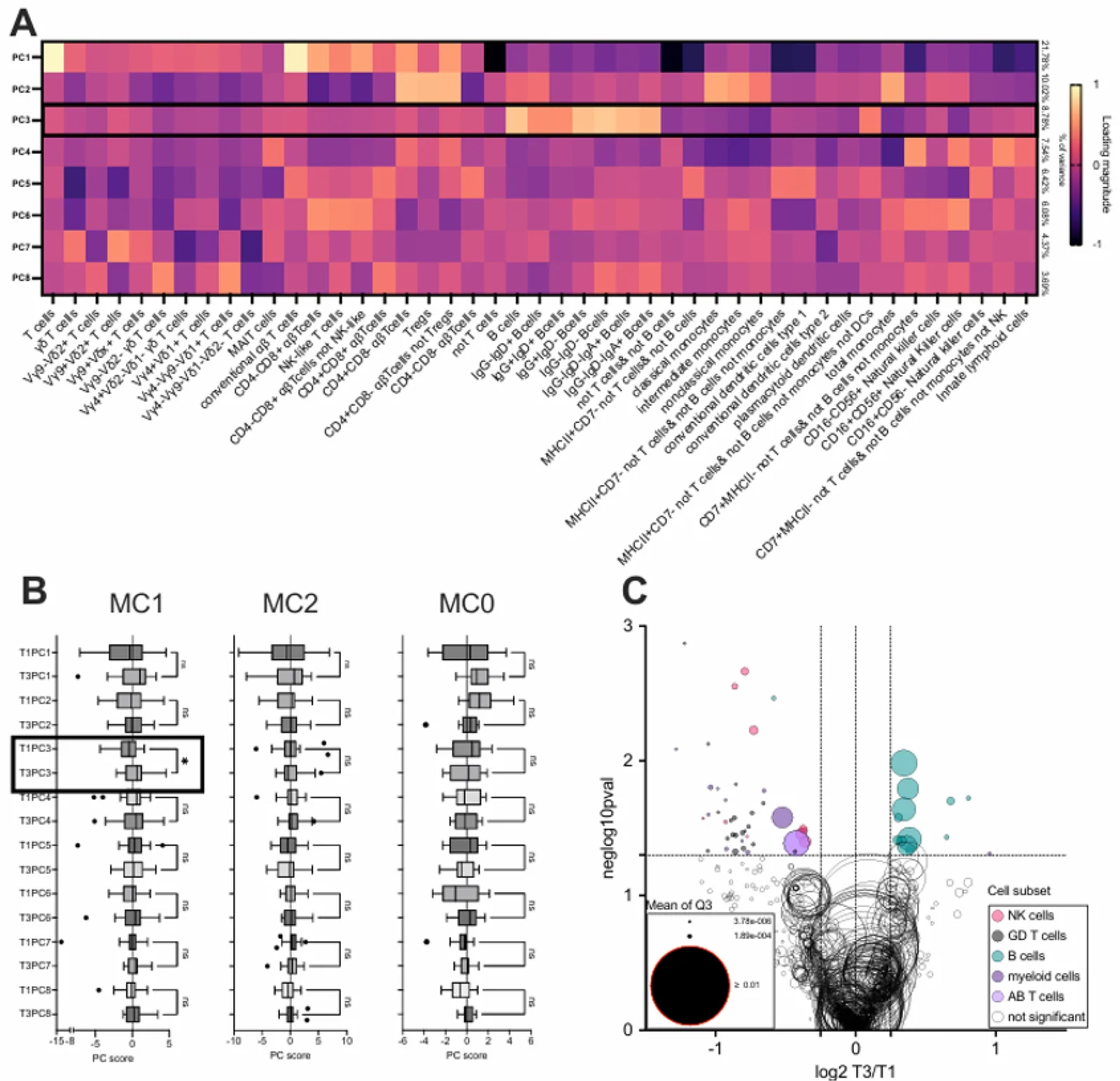
Flow cytometry-based immunophenotyping of CLBP patients’ blood. (**A**) PCA loading matrix of 8 principal components (PCs) by 45 lineage subsets of immune cells determined by multicolour flow cytometry. The loading magnitude is depicted by colour. The percentage of total variance explained by each PC is described on the right side of the loading matrix. (**B**) Comparison of PC scores for individual PCs between MC1 (**B-left**), MC2 (**B-middle**) and MC0 (**B-right**) in PA-high (tertile 3- “T3”) and PA-low (tertile 1 – “T1”) patients. (* = *P* < 0.05) (**C**) Comparison of the proportions of 1021 immune cell populations between MPA-high (T3) and PA-low (T1) patients using a generalised linear mixed effect model. The size of a symbol corresponds to the estimated size of the population in PA-High. The colour of the symbol represents the parent population. Dotted horizontal line represents negative log10 pval=1.3 (pval=0.05). The area between dotted vertical lines represents log2 fold change >−0.25 and <0.25.

PCA was used to reduce the dimensionality of the flow cytometry data (Fig. 3A). The first eight PCs cumulatively explained 68.7% of the variance in cell subset percentages, with PC1 capturing 21.8%, PC2 10.0%, and PC3 8.8% (Fig. 3A, B). When comparing PC values between patients with PA-low (PA-low) and PA-high (PA-high), PC3 values were significantly higher in patients with MC1 and PA-high (PA-high) than in those with MC1 and PA-low (PA-low) (Fig. 3B). PC3 values were weighted heavily by B cell signature (Fig. 3A). There were no other significant differences in PC values (Fig. 3B), so subsequent analyses focused on patients with MC1.

The 1021 analysed cell populations were assigned into seven major populations: B cells (127 subpopulations), all T cells (31 subpopulations), αβ T cells (276 subpopulations), γδ T cells (219 subpopulations), myeloid cells (213 subpopulations), NK cells (122 subpopulations), and ILCs (31 subpopulations) and 3 populations of mixed phenotypes (**Tab. S4**). Comparisons of all immune cell populations between PA-low and PA-high in MC1 patients revealed an increased proportion of 13 immune cell subsets (Fig. 3C, Table 2, **Tab. S4**) and a decreased proportion of 43 populations (Fig. 3C, **Tab. S4**). 12 of the 13 populations enriched in PA-high were B cells. Fisher’s exact test confirmed significant enrichment of B cell subpopulations among populations enriched in PA-high patients (p < 0.001).

**Figure 4:**
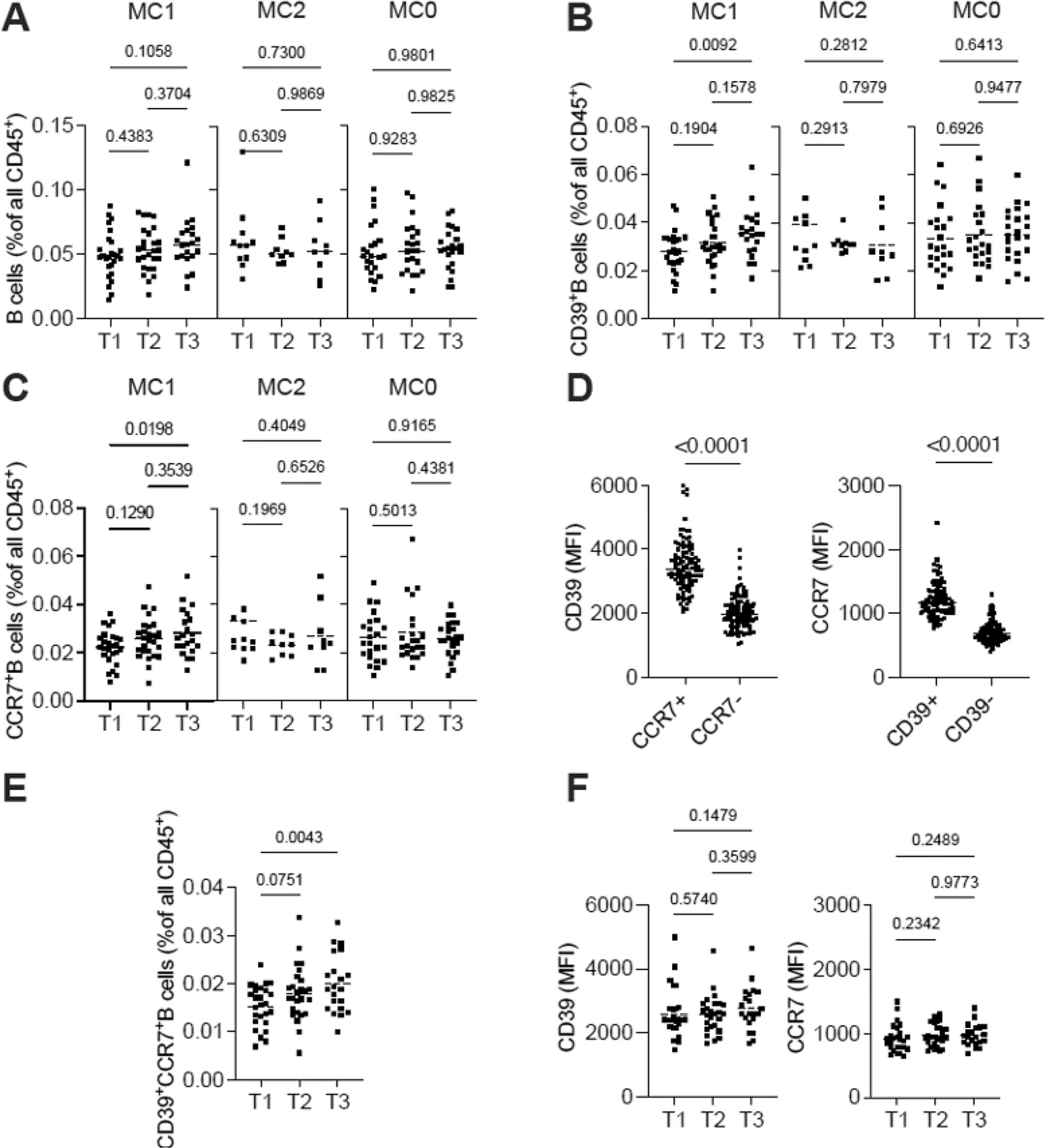
Comparison of B cell subsets between PA tertiles in patients with MC1, MC2 and MC0. Patients were grouped as PA-low (T1), intermediate PA (T2) and PA-high (T3). **(A)** The proportion of B cells; (**B**) the proportion of CD39^+^B cells; and (**C**) the proportion of CCR7^+^B cells. (**D**) Comparison of CD39 expression on CCR7^+^ and CCR7^−^ B cells (left) and CCR7 expression on CD39^+^ and CD39^−^ B cells (right). (**E**) Comparison of the proportion of CD39^+^CCR7^+^B cells between patients with PA-low (T1), intermediate PA (T2) and PA-high (T3) for patients with MC1. (**F**) Comparison of the median fluorescence intensity of CD39 (left) and CCR7 (right) on B cells from patients with MC1 and PA-low (T1), intermediate PA (T2), and PA-high (T3).

Enrichment of specific B cell subsets in PA-high MC1 patients led us to investigate whether the degree of enrichment correlates with intradiscal PA levels, and if a similar enrichment is observed in patients with MC2 and MC0. There were no significant differences in the proportion of B cells between patients in each PA tertile for all MC types (Fig. 4A). Out of twelve B cell subsets enriched in PA-high MC1 patients (**Tab. S4**), two were covering total B cells expressing one specific surface marker – CCR7^+^ and CD39^+^ B cells. Their analysis revealed that CD39^+^ B cells (Fig. 4B) and CCR7^+^ B cells (Fig. 4C) proportions were significantly higher amongst patients with MC1 and PA-high vs. PA-low, but not different amongst patients with MC2 or MC0. In addition, B cell activation markers were increased in patients with higher intradiscal PA levels. Specifically, there was a significant linear trend for an increase in proportions of CD39^+^ B cells (p = 0.009, slope = 0.0036) and proportions of CCR7^+^ B cells (p = 0.018, slope = 0.0029) with increasing PA level (from PA-low to PA-high) in patients with MC1. Comparing CD39 expression on CCR7^+^ and CCR7^−^ B cells and CCR7 expression on CD39^+^ and CD39^−^ B cells revealed that B cells expressing either of these markers also expressed significantly higher levels of the other marker (Fig. 4D), indicating that they are expressed by the same B cell population. Consequently, the proportion of CCR7^+^CD39^+^ B cells was markedly different between patients with MC1 and PA-high vs. PA-low (Fig. 4E). Lastly, we found no difference in median fluorescence intensity (MFI) of CD39 and CCR7 on B cells between PA tertiles, indicating these findings reflect differences in the relative abundance of CCR7^+^CD39^+^ B cells and not differences in the expression levels of those markers (Fig. 4F).

**Figure 5:**
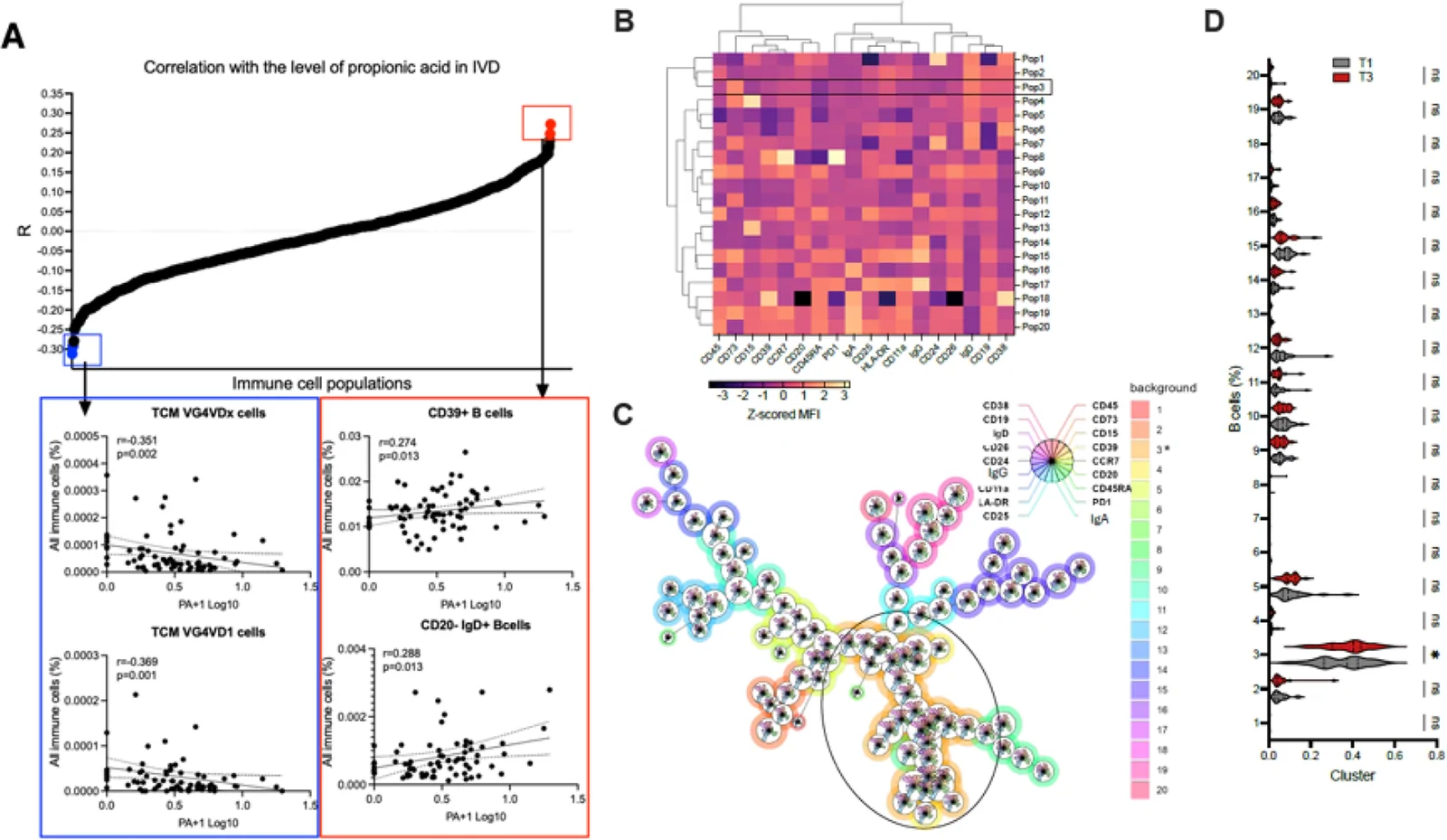
Correlation of intradiscal propionic acid with immune cell subsets and B cell subclustering. **(A)** Spearman correlation between PA level and immune cell subpopulation in MC1 patients (top). Correlations for the two immune cell populations with the greatest negative Spearman coefficient (blue) and the strongest positive Spearman coefficient (red) are given in the bottom panels. **(B)** Flow Self Organising Map (FlowSOM) clustering of B cells followed by hierarchical clustering. **(C)** FlowSOM minimal spanning tree visualisation of clustered cytometry data. Each node represents a cluster (SOM node) containing similar cells based on marker expression. The size of the nodes corresponds to the number of cells in the cluster. Edges represent similarity between clusters. Node background colours indicate meta-cluster assignment as specified in the legend. Nodes with similar profiles are connected to form a tree structure that preserves local similarity in the high-dimensional space. **D.)**Comparison of FlowSOM meta-clusters using two-way Analysis of Variance revealed similar B cell meta-clusters in patients with PA-high (T3) vs. PA-low (T1). Metacluster 3 was significantly higher in patients with PA-high (T3). (* = *P*< 0.05).

Investigation of the correlation between intradiscal PA values and the proportion of various immune cell subsets revealed significant negative correlations with PA for 44 subsets and significant positive correlations with PA for two subsets (Fig. 5A, **Tab. S5**). CD39^+^ B cells were one of only two subsets significantly positively correlated with intradiscal PA levels (ρ = 0.274, *p* = 0.013), whereas TCM Vγ4δx T cells showed one of the strongest negative correlations (ρ =−0.351, *p* = 0.002) (Fig. 5A). Since B cells were the most significantly positively correlated with intradiscal PA levels, we investigated differences within the B cell population using unsupervised clustering by FlowSOM algorithm (Fig. 5B–D). Clustering was performed based on the expression of all stained markers relevant for B cells (CCR7, CD11a, CD15, CD19, CD20, CD24, CD25, CD26, CD38, CD39, CD45, CD45RA, CD73, HLA-DR, IgA, IgD, IgG, PD1) (Fig. 5B–C). Using a 2-way ANOVA, we observed no overall effect of intradiscal PA level on B cell composition; however, there was a significant enrichment in one specific population, represented by metacluster 3 (Fig. 5D). This cluster consisted of IgD^+^ B cells with high surface expression of CD73 (Fig. 5B).

### Serum cytokines

**Figure 6:**
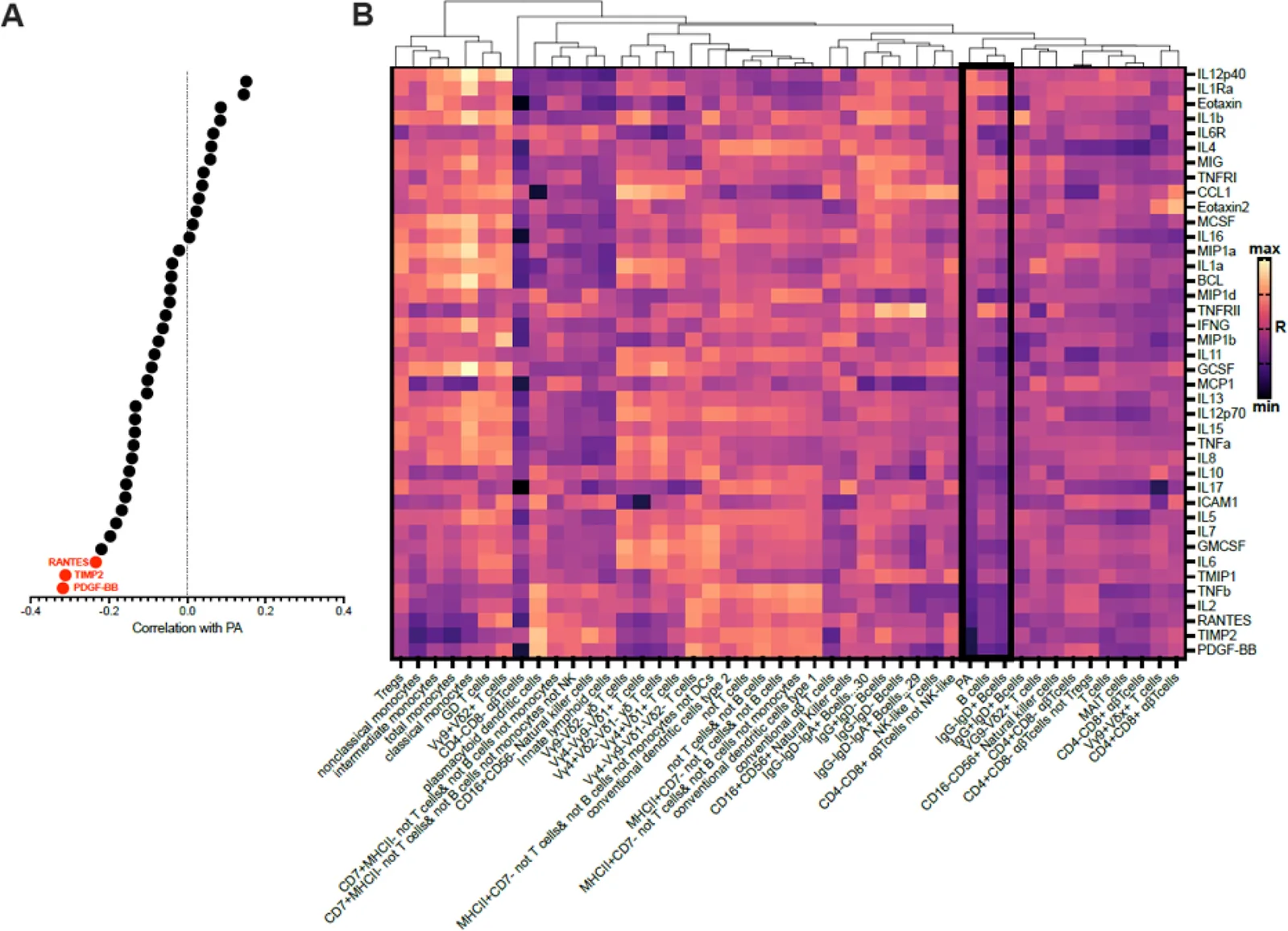
Serum cytokine correlations with intradiscal propionic acid and immune cell populations in MC1. **A.)** Spearman correlation coefficients between PA level and blood serum cytokines. Cytokines with significant correlations are highlighted in red. **B.)** Heatmap depicting Spearman correlation coefficients of major immune cell populations and intradiscal PA with blood plasma cytokines in patients with MC1. Rows are organised based on cytokine correlations with intradiscal PA, from highest to lowest. Columns are organised by hierarchical agglomerative clustering using Euclidean distance and complete linkage. Thick black framing is used to depict the column corresponding to intradiscal PA and the closest clustered immune cell populations – B cells and IgD^+^ B cells.

Serum samples from 398 patients were assayed for forty cytokines associated with inflammation. Out of 15,920 total cytokine values, 860 missing values (5.4%) were imputed. Among these were 73 MC1, 29 MC2 and 68 MC0 patients, for whom we also performed MRS-based PA quantification and flow cytometry immunophenotyping. Spearman correlation of blood serum cytokine levels with PA MC1 patients revealed a significant negative correlation between PDGF-BB (ρ =−0.32, p = 0.007), TIMP2 (ρ =−0.31, p = 0.008), RANTES (ρ =−0.23, p = 0.049) and PA levels (Fig. 6A, **Tab. S6**). No significant positive correlation was detected. The highest correlation coefficient was observed for IL12-p40 (ρ = 0.15, p = 0.21). Markedly different results were obtained for MC2 and MC0, with IL-12-p40 and IL-4 showing a significant negative correlation with PA in both (**Fig. S6**). In MC2, a significant negative correlation was also observed with TNFRI, and in MC0, with M-CSF and G-CSF (**Fig. S6**). Hierarchical clustering of correlation coefficients between immune cell subsets and PA and cytokine levels in MC1 patients, using Euclidean distance and agglomerative complete-linkage clustering, revealed that correlations of cytokines with PA were closest to those with B cells and IgD^+^ B cells (Fig. 6B, **Tab. S7**). In MC0 and MC2 patients, PA levels correlate most closely with plasma cytokines associated with NK cells and nonclassical monocytes **(Fig. S7**).

## Discussion

This study provides convergent evidence from transcriptomic, multiparametric flow cytometry, and serum cytokine analysis showing that intradiscal PA levels are associated with both the relative abundance and activation state of peripheral B cells in patients with MC1, the MC subtype most consistently associated with pain^34^. Notably, these B cell responses were not observed in patients with MC2 or in those without MC.

Our transcriptome analysis revealed pronounced enrichment of gene sets related to adaptive immune responses and B cell activation in PA-high MC1 cases, including upregulation of immunoglobulin heavy chain transcripts and pathways such as “positive regulation of B cell activation” and “B cell receptor signalling”. Flow cytometry further demonstrated that amongst the more than one thousand immune subsets analysed, B cells – particularly those expressing activation markers CCR7 and CD39, and most significantly the CCR7^+^CD39^+^ double-positive subset – were enriched in PA-high MC1^35,36^. Data-driven unsupervised clustering independently highlighted an IgD^+^CD73^+^ B cell metacluster enriched in these patients. These findings indicate that both hypothesis-driven and data-driven strategies consistently converge on B cells as the central immune cell population associated with increased intradiscal PA in MC1.

Cytokine profiling showed that in MC1 patients, PA levels were inversely correlated with serum concentrations of PDGF-BB, TIMP2, and RANTES – factors involved in tissue repair, extracellular matrix modulation, and immune cell recruitment^37–39^. Interestingly, the pattern of PA correlation with cytokines was markedly different in MC1 patients than in MC2 and MC0. In addition, in MC1, hierarchical clustering aligned PA-cytokine correlation patterns closely with cytokine correlation with B and IgD^+^ B cells, which was not observed for MC2 and MC0 patients. These patterns together add an additional layer of support for the association between B cells and intradiscal PA levels in MC1 patients.

### Mechanistic Connections Between Propionic Acid and B Cells

The observed association between PA and B cell profiles might reflect: i.) the direct effects of PA on B cells; and/or ii.) the adaptive immune response to *C. acnes* as a PA source. iii.) the autoimmune response to propionylated proteins.

#### i.) PA as an immunomodulator

Short-chain fatty acids, including propionic acid, are known primarily for their immunosuppressive potential, which plays an important role especially in organs with high concentrations of these bacterial metabolites such as the intestine^40^. PA has been shown to modulate both systemic and tissue-resident immunity by directly influencing B cell function and phenotype^41^. In a murine model and in vitro cultures of human B cells, PA can stabilise IL-10 expression in B cells, promoting a regulatory phenotype and supporting immune tolerance^41^. PA can also suppress activation of the toll-like-receptor (TLR)4 / NF-KB pathway and has a rather regenerative than pro-inflammatory effect^42,43^. This might be relevant in MC1 pathobiology because discs at MC1 levels contain more TLR4 agonists^9^. However, the effects of PA are highly context- and cell-type-dependent, and PA can act as both a regulator and a potentiator of inflammation^44^. PA, when released in high concentrations during bacterial fermentation in lipid-rich, hypoxic environments, strongly inhibits HDAC8 and HDAC9 in human epithelial keratinocytes, leading to increased histone acetylation at the promoters of inflammatory genes. This epigenetic mechanism substantially enhances the expression of proinflammatory cytokines (such as IL-6, IL-8, and CCL5/RANTES) in keratinocytes following TLR stimulation, contrasting the anti-inflammatory effects of HDAC inhibition in monocytes and other immune cells^44^. Notably, the hypoxic intervertebral disc microenvironment mirrors the conditions under which these pro-inflammatory epigenetic effects were observed, suggesting that disc and cartilage endplate cells respond to PA more like keratinocytes than circulating immune cells. Whether PA amplifies local inflammatory signaling in MC1 warrants future experimental investigation of PA effects on individual cell types residing in human IVDs.

#### ii.) Immune response to anaerobic bacteria as a PA source

An alternative explanation is that PA serves primarily as a surrogate marker for the presence and activity of bacteria residing in the hypoxic environment of intervertebral discs, such as *C. acnes*. *C. acnes* has been consistently found in intervertebral discs, and it is known to produce PA as a major metabolic byproduct^18,45^. In this model, elevated PA, as measured by MRS, reflects a higher bacterial burden, and the observed enrichment of B cell subsets in PA-high MC1 patients represents an adaptive immune response against bacterial antigens. Whether such B cell responses are protective by contributing to bacterial clearance, or pathologic by sustaining inflammation and pain, remains unresolved. The model of immune response against *C. acnes* is further supported by the transcriptomic signature of “Defence response to bacterium” and enrichment of neutrophil-related genes. *C. acnes* has been shown to trigger inflammation in discs, and neutrophils were activated in MC1 bone marrow adjacent to discs with high *C. acnes* loads^10,46^. It should be noted that *C. acnes*-associated MC1 are distinct from spondylodiscitis^47^.

We observe that the B cells accumulating in PA-high MC1 patients are often CCR7^+^, CD39^+^ and CD73^+^, which suggests their activation^35,36^. However, the expression of CD39 and CD73 can also be associated with PA’s immunomodulatory effects, as CD39 and CD73 are key ectonucleotidases involved in the generation of extracellular adenosine, a molecule with anti-inflammatory properties^48,49^. Recent studies, for example, showed that PA exposure enhances the suppressive capacity of regulatory B cells and increases IL-10 and TGF-β expression, linking microbial metabolite sensing to the control of autoimmunity and inflammation^41^. These immunoregulatory B cell subsets have been implicated in a variety of chronic inflammatory and autoimmune diseases^50^. It thus remains to be determined whether the B cells accumulating in MC1 are primarily protective or pathogenic.

#### iii. Immune response to propionylated proteins

Human proteins might be reversibly modified by propionylation of the -amino group of lysine residues^51^. Propionylation changes lysine charge from + 1 to 0, modifying physical properties of proteins^52^. These structural changes may expose or generate neoepitopes capable of eliciting immune responses, as is known for citrullinated peptides in rheumatoid arthritis^53,54^. Whether antibodies against propionylated proteins occur in patients with MC1 was not addressed in this study and remains to be determined.

### Translational Relevance and Future Directions

Identifying B cells as central immunological mediators in MC1 raises the possibility of repurposing or developing B cell-directed therapies for patients with MC1-related back pain and PA-high, mirroring advances made in other immune-mediated musculoskeletal diseases such as rheumatoid arthritis^55–57^. The observed B cell signature may also serve as a biomarker for *C. acnes*-caused MC1 and to stratify MC1 patients for antibiotic therapy, in case future studies can link high disc PA to high *C. acnes* loads. Further mechanistic studies are warranted to elucidate the antigenic specificity and functional potential of these B cell subsets, as well as to develop experimental models that directly test the modulatory role of PA in chronic discogenic pain.

### Limitations of the Study

The main limitation of this observational study is that disc tissues were unavailable for analysis. Thus, we did not measure the amount of intradiscal *C. acnes* or directly validate the concentration of intradiscal PA. Therefore, it cannot be fully excluded that PA measurement might reflect past infection, non-viable bacterial metabolites, or non-microbial metabolic processes. We previously found that *C. acnes-* and PA-injected bovine discs had clear MRS peaks corresponding to the chemical shift of PA (~ 1.05 ppm), whereas uninjected discs lacked the characteristic peak^18^. Also, the signal intensity at this characteristic chemical shift increased with increasing volume of *C. acnes* in the injectate. While these findings support the sensitivity and specificity of MRS, we cannot exclude the possibility that other sources of PA contributed to the MRS signal in this study. Furthermore, we performed an associative analysis, which limits interpretation due to potential confounding and selection bias.

An additional limitation of the study is that differences in circulating inflammatory factors or cell populations may reflect underlying comorbidities unrelated to CLBP status. Patients were excluded from the study if they were pregnant or had known conditions that affect systemic immune markers, e.g., ankylosing spondylitis, rheumatoid arthritis, polymyalgia rheumatica, psoriatic arthritis, lupus, cancer, discitis, or osteomyelitis^28^. However, we cannot exclude the possibility that some patients had undiagnosed conditions or infections that influenced the composition and activation of the immune system

## Conclusions

This study establishes B cell activation as a core immune correlate of high intradiscal PA in LBP patients with lumbar MC1. Our findings indicate that local PA changes in the disc are linked to systemically detectable changes of the adaptive immune system in MC1 patients, suggesting that MC1 has a systemic immune component. This supports our recent findings of HLA association with MC1^58^. These findings advance our understanding of the immunometabolic interface in spine disease and support a personalised biomarker- and cell-targeted therapeutic approach for MC1-related LBP.

## Supplementary Material

Supplementary Files

This is a list of supplementary files associated with this preprint. Click to download.

- FigureS1.pdf
- FigureS4.pdf
- FigureS3.pdf
- FigureS6.pdf
- FigureS2.pdf
- FigureS7.pdf
- FigureS5.pdf
- TableS4.xlsx
- TableS1.xlsx
- TableS3.xlsx
- TableS7.xlsx
- TableS6.xlsx
- TableS5.xlsx
- TableS2.xlsx

## Figures and Tables

**Table 1:** Demographics and clinical characteristics of LBP patients.

Characteristic	Modic change				
	Total (*n* = 200)	MC0 (*n* = 85)	MC1 (*n* = 80)	MC2 (*n* = 35)	*p*-value
Demographics					
Age (year)	51.3 ± 15.6	45.8 ± 16.8^a^	54.5 ± 13.4^b^	57.2 ± 13.0^b^	< 0.0001***
Male	85 (42.5)	31 (36.5)	39 (48.8)	15 (42.9)	0.39
BMI (kg/m^2^)	26.2 ± 4.7	25.4 ± 4.1^a^	26.0 ± 3.9^a^	28.5 ± 6.5^b^	0.003**
Clinical measures					
PEG	4.5 ± 1.9	4.4 ± 1.8	4.6 ± 1.9	4.4 ± 1.7	0.84
PROMIS PI	58.5 ± 7.7	58.6 ± 6.8	57.8 ± 8.3	59.9 ± 8.4	0.42
PROMIS PF	41.8 ± 6.4	41.7 ± 5.6	42.3 ± 6.9	40.9 ± 7.0	0.56
PA	3.2 ± 3.2	3.2 ± 3.0	3.1 ± 3.4	3.4 ± 3.1	0.86

** *p* < 0.01,

*** *p* < 0.001

For continuous numerical variables, mean values ± standard deviations are indicated. Overall group differences between MC types were tested with one-way ANOVA. For count data (Male), the count is indicated, and the percentage is in parentheses. Pairwise comparisons using Tukey-Kramer HSD were performed for asterisked characteristics, and connecting groups are marked with superscripts (a, b); all have a significance of *p* < 0.05. BMI: body mass index; PEG: pain scale (0–10); PROMIS PI: Patient-Reported Outcomes Measurement Information System pain interference T-score; PROMIS PF: physical function T-score; PA: maximum measured intradiscal PA.

**Table 2:** Differentially abundant cell subsets between patients with “PA-high” vs. “PA-low” and MC1.

Major cell population	Cell subset	Fold-change	*p*-value
Myeloid cells	CD11b^−^ intermediate monocytes	1.94	0.049
B cells	IgG^+^CD123^+^	1.75	0.019
B cells	IgD^+^CD20^−^	1.60	0.020
B cells	IgA^+^HLADR^−^	1.57	0.037
B cells	IgX^+^CD24^−^	1.31	0.046
B cells	CD24^−^	1.31	0.038
B cells	IgD^+^CD39^+^	1.29	0.016
B cells	IgD^+^CCR7^+^	1.29	0.042
B cells	CCR7^+^	1.27	0.023
B cells	CD39^+^	1.27	0.010
B cells	IgX^+^CD15^−^	1.25	0.040
B cells	IgX^+^CCR7^+^	1.23	0.026
B cells	IgX^+^CD39^+^	1.23	0.039

## Data Availability

All data are available upon reasonable request. The whole-blood transcriptomic data will be deposited in a public repository, and the accession number will be provided during peer review.

## Abbreviations

ANOVA: analysis of variance
AUC: area under the curve
BMI: body mass index
CHESS: chemical selective suppression
CLBP: chronic low back pain
DEG: differentially expressed gene
ELISA: enzyme-linked immunosorbent assay
FDR: false discovery rate
FlowSOM: flow self-organising map
GO: Gene Ontology
GSEA: gene-set enrichment analysis
HDAC: histone deacetylase
IVD: intervertebral disc
LBP: low back pain
LOD: limit of detection
MC: Modic changes
MC0: no Modic changes
MC1: Modic type 1 changes
MC2: Modic type 2 changes
MC3: Modic type 3 changes
MFI: median fluorescence intensity
MRI: magnetic resonance imaging
MRS: magnetic resonance spectroscopy
NK: natural killer
PA: propionic acid
PC: principal component
PCA: principal component analysis
PEG: pain,enjoyment of life and general activity scale
PF: physical function
PI: pain interference
PRESS: point-resolved spectroscopy
PROMIS: Patient-Reported Outcomes Measurement Information System
RIN: RNA integrity number
RPMI: Roswell Park Memorial Institute medium
SD: standard deviation
TCM: central memory T cell
TLR: toll-like receptor
